# Real-Time Analysis of Isoprene in Breath by Using Ultraviolet-Absorption Spectroscopy with a Hollow Optical Fiber Gas Cell

**DOI:** 10.3390/s16122058

**Published:** 2016-12-05

**Authors:** Takuro Iwata, Takashi Katagiri, Yuji Matsuura

**Affiliations:** 1 Graduate School of Biomedical Engineering, Tohoku University, 6-6-05 Aoba, Sendai 980-8579, Japan; takuro.iwata.s8@dc.tohoku.ac.jp; 2 Graduate School of Engineering, Tohoku University, 6-6-05 Aoba, Sendai 980-8579, Japan; katagiri@ecei.tohoku.ac.jp

**Keywords:** breath analysis, isoprene, ultraviolet spectroscopy, hollow-optical fiber

## Abstract

A breath analysis system based on ultraviolet-absorption spectroscopy was developed by using a hollow optical fiber as a gas cell for real-time monitoring of isoprene in breath. The hollow optical fiber functions as an ultra-small-volume gas cell with a long path. The measurement sensitivity of the system was evaluated by using nitric-oxide gas as a gas sample. The evaluation result showed that the developed system, using a laser-driven, high-intensity light source and a 3-m-long, aluminum-coated hollow optical fiber, could successfully measure nitric-oxide gas with a 50 ppb concentration. An absorption spectrum of a breath sample in the wavelength region of around 200–300 nm was measured, and the measured spectrum revealed the main absorbing components in breath as water vapor, isoprene, and ozone converted from oxygen by radiation of ultraviolet light. The concentration of isoprene in breath was estimated by multiple linear regression. The regression analysis results showed that the proposed analysis system enables real-time monitoring of isoprene during the exhaling of breath. Accordingly, it is suitable for measuring the circadian variation of isoprene.

## 1. Introduction

Breath analysis has attracted a lot of attention because it can noninvasively monitor the early stages of disease states [1,2]. Besides major compounds such as nitrogen, oxygen, carbon dioxide, and water, human breath gas contains various components, including inorganic gases such as nitric monoxide and carbon monoxide [3,4], and volatile organic compounds such as acetone and isoprene [5], and it is known that these minor components reflect the internal metabolism. To analyze these minor components quantitatively, a measurement system with very high sensitivity is necessary because their concentrations are usually as low as 10 to 500 ppb. For this reason, large-scale gas chromatography–mass spectrometry (GC-MS) systems have been commonly used for the analysis of such trace gases [6,7,8]. However, these systems are usually bulky and high-cost; therefore, it is difficult to practically use them in hospitals and clinics. To overcome these problems, optical analysis methods have been of interest because they usually have an advantage over GC-MS methods in terms of system cost. In addition, with optical analysis methods, samples can be measured without the sample treatment or preparation that is needed in the case of GC-MS methods [9,10].

For breath analysis, a variety of optical analysis methods have been proposed. One, based on Raman spectroscopy, utilized hollow-core photonic crystal fibers to enhance Raman signals and obtain high sensitivities [11,12]. Another method, based on photoacoustics, detected the optical absorption of a sample in the near-infrared (NIR) or mid-infrared (MIR) regions [13]. As for MIR absorption spectroscopy, the sensitivity was enhanced by using optical cavities as gas cells [14]. More recently, a compact cavity-enhanced spectroscopy system was developed by using quantum-cascade lasers emitting MIR light [15].

Analyzing breath by absorption spectroscopy using an ultraviolet (UV) light source (which detects volatile gases showing relatively large absorption in the UV region) has an advantage over MIR spectroscopy because it is less affected by the water vapor in breath. In common MIR spectroscopy, a desiccant such as silica gel, molecular sieves, or zeolite is usually necessary because water vapor in breath exhibits strong absorption in the MIR region. However, a desiccant also absorbs some minor components other than water, so using a desiccant sometimes causes problems. The sensitivity of UV spectroscopy was reportedly improved by introducing an optical cavity cell [16,17], although the structure of the system would be somewhat complicated and the system cost tends to be high. Human breath can be analyzed by using a simple UV spectrometer with a deuterium (D_2_) lamp and a light-pipe gas cell. For instance, Baum et al. showed that a UV spectroscopic system is useful for monitoring cardiac output by a technique called acetylene-based, foreign-gas rebreathing [18]. However, its sensitivity was not good enough for detecting intrinsic minor components in breath.

We have previously proposed using hollow optical fibers composed of glass capillaries (with a metal and dielectric coating on the inside) as a gas cell. This hollow fiber gas cell provides a long optical path with extremely small volume, and we showed its usefulness for MIR spectroscopy [19] and Raman spectroscopy [20]. It was also shown that a spectroscopy system based on a hollow optical fiber gas cell enhances sensitivity of UV spectroscopy for gas analysis [21,22].

In the current study, a simple breath-analysis system based on UV-absorption spectroscopy with a hollow-optical-fiber gas cell is proposed. This system achieves highly sensitive measurement at low cost owing to its high-intensity, broad UV light source and hollow optical fiber gas cell. Its sensitivity is improved by using long hollow fiber cells so that it can quantitatively measure the isoprene concentration in human breath.

## 2. Ultraviolet-Absorption Spectroscopy

**Figure 1:**
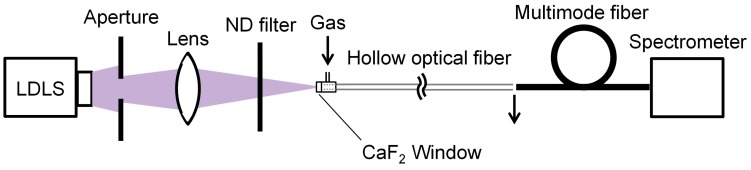
Experimental setup for UV-absorption spectroscopy of gases.

A schematic of the experimental setup for UV-absorption spectroscopy of gases is shown in Figure 1. As a light source, a laser-driven UV light (LDLS, EQ-99N, Energetiq Technology, Inc., Woburn, MA, USA) was used because it was expected to attain a higher signal-to-noise ratio (SNR) owing to the higher intensity of the emitted UV light than is possible with common deuterium lamps. The LDLS emitted light with a wavelength from 170 to 2100 nm and the output light was focused on a hollow optical fiber with an inner diameter of 1 mm by using a CaF_2_ lens with a 100 mm focal length. An aperture was set between the light source and the lens to make a focal spot that fit the bore of the hollow optical fiber. A neutral density filter was inserted in the light path to stop the light intensity from being saturated. Although the fiber was kept straight in our experiment, it could be looped with a bending radius of around 30 cm. We used an aluminum-coated hollow optical fiber (UVS1000, Doko Engineering LLC, Sendai, Miyagi, Japan) as a gas cell. The base material of the hollow optical fiber is a silica-glass capillary, the inner surface of which is coated with an aluminum film (exhibiting high reflection in the UV region [23,24]) so that the fiber has a low transmission loss for UV light. The incident end of the fiber was sealed by a metal sleeve with a CaF_2_ window and a gas inlet. The sample gases were introduced into the hollow core of the fiber via a gap between the window and the fiber’s end surface. The gap was set to less than 1 mm so as not to cause a dead volume for the sample gases. The output end was open to make the gas flow out and a multimode silica-glass fiber with a core diameter of 600 μm was connected to the output end of the hollow fiber to deliver the output light to a conventional fiber-optic spectrometer (C10082CA, Hamamatsu Photonics K. K., Hamamatsu, Shizuoka, Japan) for measuring the output power spectra. The detection wavelength range of the spectrometer was 200 to 800 nm and the wavelength resolution was 3.2 nm in the UV region.

**Figure 2:**
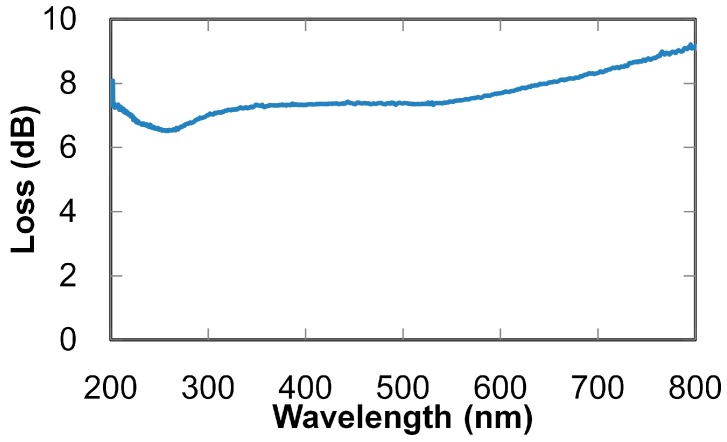
Transmission-loss spectrum of aluminum-coated hollow optical fiber with inner diameter of 1 mm and length of 1 m.

**Figure 3:**
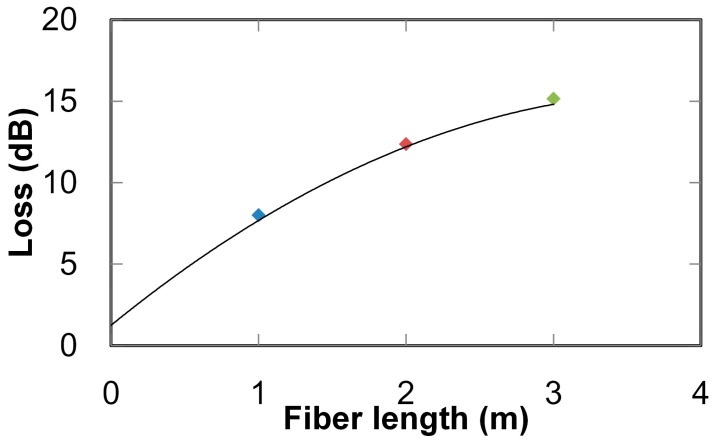
Transmission losses of aluminum hollow optical fiber with different lengths measured at wavelength of 200 nm.

A transmission-loss spectrum of the aluminum hollow optical fiber (with an inner diameter of 1 mm and a length of 1 m) is shown in Figure 2. The figure demonstrates that the fiber shows broadly low losses in the UV to near-infrared regions (i.e., 200 to 800 nm), so the losses for UV light emitted from the LDLS are considered to be low. Relations between the fiber length and transmission loss measured at a wavelength of 200 nm are shown in Figure 3. Transmission loss does not linearly increase with the fiber length because high-order modes (exhibiting relatively high losses) attenuate when light is transmitting in the hollow-optical fiber, so only low-order modes with low losses survive in long fibers. This result revealed that the loss of the 3-m-long fiber was approximately 16 dB, and from the intercept of the curve fitted to the measured results, the coupling loss between the light source and the fiber was estimated to be around 1.3 dB.

**Figure 4:**
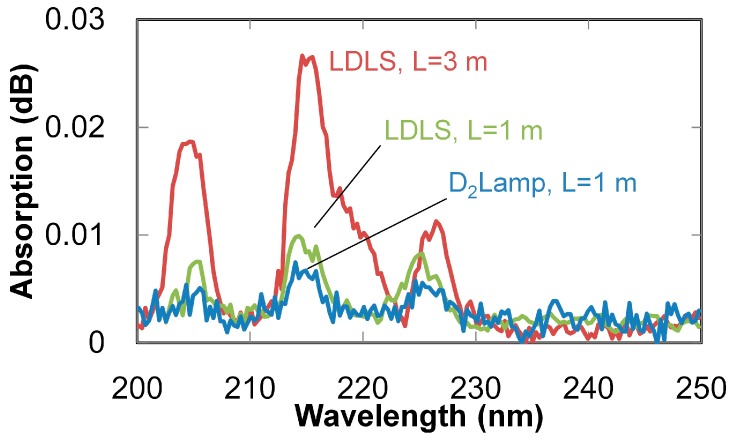
Absorption spectra of NO gases (at 1 ppm) measured by three different setups: (i) LDLS light source with 3-m-long fiber; (ii) LDLS with 1-m-long fiber; and (iii) deuterium lamp source with 1-m-long fiber.

To evaluate the performance of the proposed system for ultraviolet-absorption spectroscopy using a hollow optical fiber, the UV absorption spectra of nitric oxide (NO) gas (which is known as a biomarker of asthma [10]) were firstly measured. Absorption spectra of sample gases were calculated from transmission-power spectra of the fiber containing a sample gas and a nitrogen background gas. Absorption spectra of 1 ppm NO gas (measured by 1- and 3-m-long hollow optical fibers) are shown in Figure 4. For comparison, an absorption spectrum measured with a common deuterium lamp and a 1 m hollow optical fiber is also shown. Absorption peaks of NO gas were clearly observed at 204, 214, and 225 nm, as previously reported [22]. Moreover, the noise level was reduced by using the LDLS, and the peak intensity was increased by the long hollow fiber. SNRs calculated from the spectra shown in Figure 4 are listed in Table 1. In the calculation, peak heights at 214 nm were used as the signal intensity, and noise amplitudes in the range of 230 to 250 nm were used as the noise intensity. These results indicate that that SNR was greatly improved by using the system with LDLS and a 3-m-long fiber.

**Figure 5:**
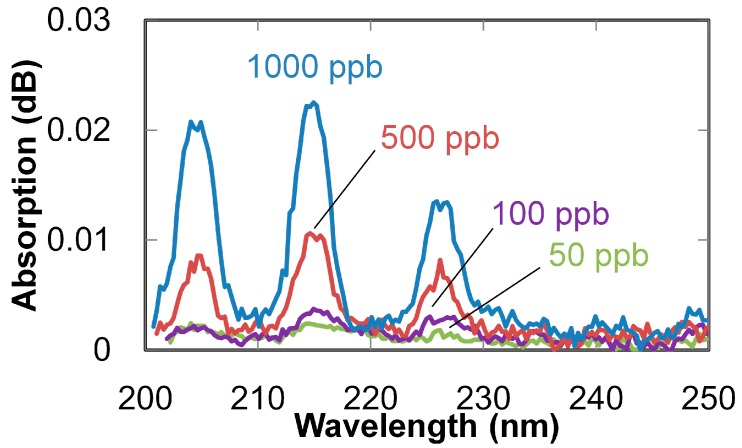
Absorption spectra of NO gases with different concentrations measured with 3 m hollow optical fiber and LDLS.

**Figure 6:**
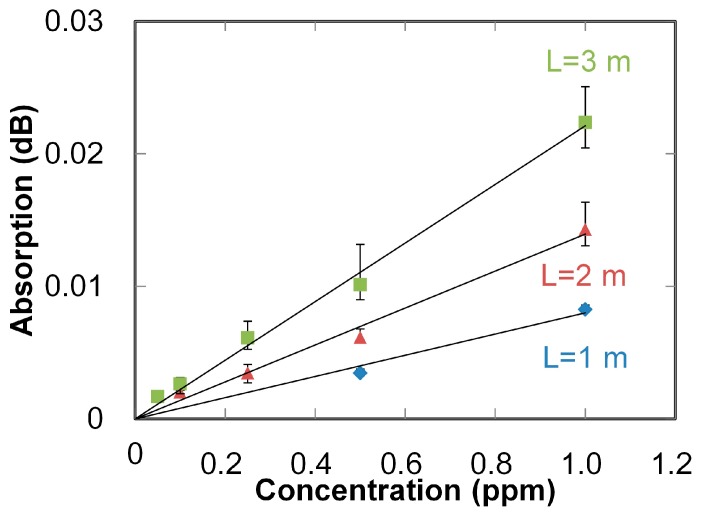
Correlations between absorptions of NO sample gases measured at 215 nm and NO gas concentration measured with different fiber lengths.

Absorption spectra of NO gases (with concentrations of 50 to 1000 ppb) measured with a 3-m-long hollow optical fiber are shown in Figure 5. Absorption peaks are clearly observed even for the lowest concentration (50 ppb). Correlation lines between the gas concentration and measured absorption at 214 nm are shown in Figure 6. The error bars in the figure show the variation of the three measurements, and the dots show the mean values. Sensitivity (which corresponds to the slope of the lines) was improved three times by elongating the fiber from 1 to 3 m; as a result, the correlation coefficient was improved from 0.990 to 0.995. The minimum detectable concentration was improved to 50 ppb from 1000 ppb, which was obtained in our previous study [25] using the combination of a common D_2_ lamp and a 1 m hollow optical fiber.

## 3. Breath Analysis

**Figure 7:**
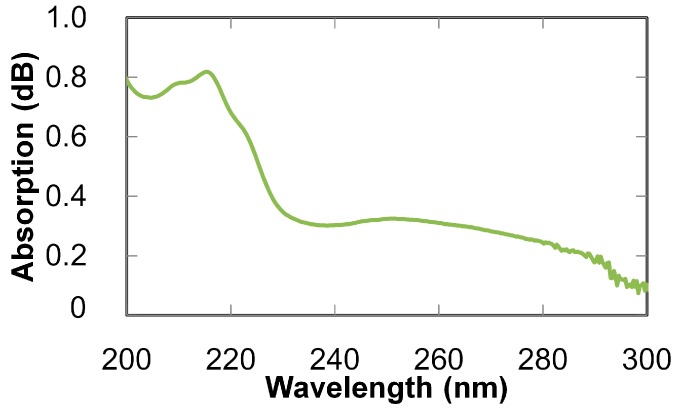
Absorption spectrum of human breath measured with 3-m-long fiber and LDLS.

**Figure 8:**
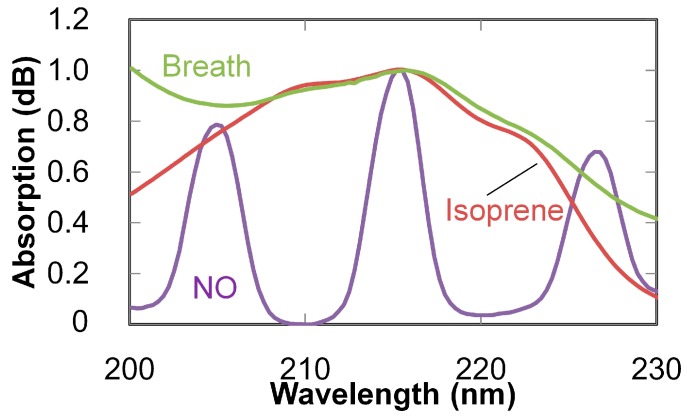
Absorption spectrum of breath (with effect of ozone removed). Absorption spectra of nitric monoxide and isoprene (normalized by peak intensities) are also shown for comparison.

Absorption spectra of human breath were measured using the setup shown in Figure 1, in which a subject exhaled into a silicone tube connected to the input end of the fiber. Absorption spectra were measured at the end of exhalation (because concentrations of volatile gases become high at that moment). As seen in the measured spectrum shown in Figure 7, a broad peak is observed around 255 nm, and a few absorption peaks are observed in the 210–225 nm range. The former peak originates from ozone [26] generated by irradiating oxygen in breath with the high intensity UV light. The latter peaks seem to be due to the absorption of nitric monoxide or isoprene in breath [16,27]. To focus on nitric monoxide and isoprene, a reference gas was prepared by irradiating a 19% oxygen gas (diluted with nitrogen) injected into the hollow optical fiber with UV light from the LDLS source to generate ozone. The absorption spectrum of this ozone reference gas was then measured, and it was subtracted from the breath spectrum shown in Figure 7 to remove the effect of the ozone. The obtained spectrum (zoomed in at the absorption bands of nitric monoxide and isoprene) is shown in Figure 8, and absorption spectra of those gases are also shown in the figure for comparison. The absorption spectra of the standard gases of nitric oxide and isoprene were measured and normalized by the peak intensities of each gas. The concentrations of the gases shown in Figure 8 correspond to 53.2 ppm for nitric oxide and 702 ppb for isoprene. To estimate the ratio of these components in breath, multiple linear regression analysis (using the measured absorptions of the standard gases shown in Figure 8 as explanatory variables) was applied. According to the results of the regression analysis, the estimated concentration of nitric monoxide was smaller than 10 ppb and, in contrast, isoprene had a concentration of 183 ppb. It is therefore concluded that isoprene is more dominant in the breath from the viewpoint of UV absorption. Human breath isoprene is known as an indicator of cholesterol synthesis and is a possible biomarker of hyperlipidemia [5,10].

**Figure 9:**
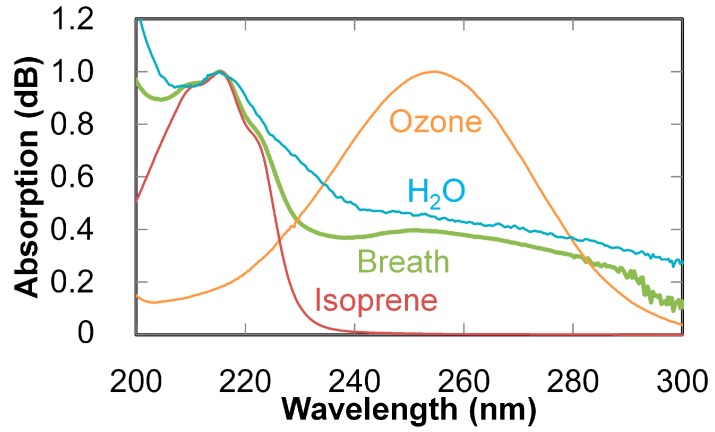
Absorption spectrum of breath (normalized with the peak intensities) compared with absorption spectra of isoprene, water vapor, and ozone.

An absorption spectrum of breath is compared with the measured absorption spectra of the standard gases of isoprene, water vapor, and ozone in Figure 9. The spectra of the standard gases were normalized by the peak intensities. The absorption peaks on the breath spectrum clearly coincide with those on the spectra of gases that are main components in breath. Multiple linear regression analysis was then applied to the breath spectrum in Figure 9 using the measured absorptions of isoprene, water vapor, and ozone as explanatory variables. According to the results of the regression analysis, the concentrations of isoprene and water were estimated as 236 ppb and 118 ppm, respectively.

**Figure 10:**
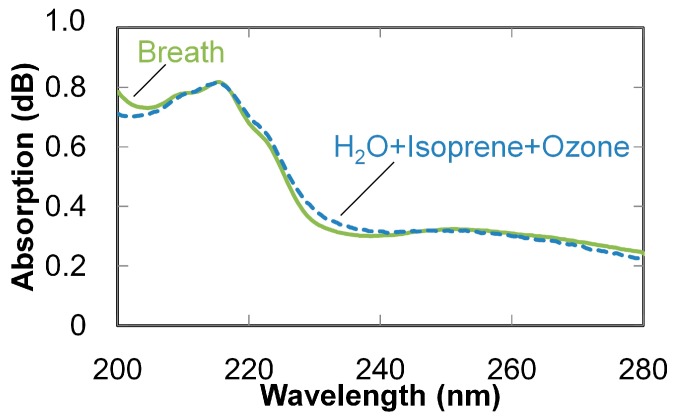
Calculated absorption spectrum of a mixed gas of isoprene, water, and ozone (whose concentrations were estimated from the measured spectrum of breath, also shown for comparison).

A measured absorption of breath is compared with a calculated absorption spectrum of a mixed gas containing isoprene, water, and ozone (whose concentrations were estimated by the multiple linear regression described above) in Figure 10. The calculated spectrum clearly coincides with the measured spectrum; therefore, it is concluded that the three components dominate the absorption spectra of breath in this UV wavelength region and that the concentrations of isoprene can be estimated from the UV absorption spectra of breath. It is known that concentrations of isoprene in breath are usually 50 to 500 ppb [28,29], which validates the concentrations estimated by our proposed system.

**Figure 11:**
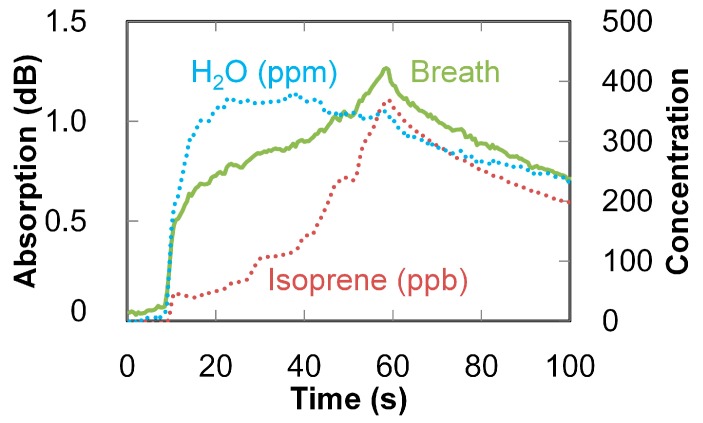
Change in the absorption at 215 nm compared with changes in concentrations of isoprene and water vapor.

We performed an experiment to show that the proposed system enables real-time monitoring of the concentration of isoprene in breath. The change in the absorption at 215 nm is compared with changes in concentrations of isoprene and water vapor in Figure 11. The concentrations were calculated by using the multiple linear regression analysis explained above. In this experiment, an examinee started slowly exhaling until almost at their limit. Then, after 50 s, they stopped blowing into the system. According to the results shown in Figure 11, water content is high at the beginning of exhaling and becomes constant. In contrast, the concentration of isoprene increases while exhaling and reaches a maximum in the final breath. This result confirms that the proposed system can quantitatively monitor the isoprene concentration in breath in real time. This performance was achieved owing to the extremely small volume (namely only 2.3 mL for the 3-m-long fiber) of the hollow fiber gas cell.

**Figure 12:**
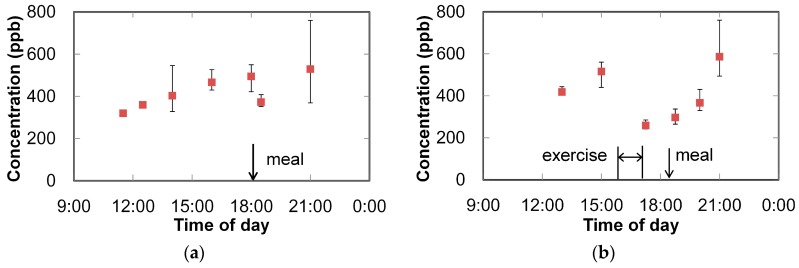
Change in measured concentration of isoprene in breath (of two examinees) during the day: (**a**) effect of eating; (**b**) effect of exercise.

Variations of the isoprene concentration in the breath of several volunteers during the day were measured, and some typical measurement results are shown in Figure 12. The error bars show the variation of the three measurements, and the square dots show the mean values. It is known that the isoprene concentration in breath decreases right after a meal because biosynthesis of cholesterol is inhibited when the digestive system takes cholesterol from foods [30]. After that, biosynthesis of cholesterol restarts, and the isoprene in breath increases until waking up in the morning. The result shown in Figure 12a reflects this tendency concerning cholesterol metabolism. The effect of exercise on cholesterol metabolism is shown in Figure 12b. During exercise, both the heart rate and breathing rate abruptly increase, resulting in a drop of the isoprene concentration in the breath [31], as shown in the figure. In both cases, the errors were large at 9 p.m. because the isoprene concentration rapidly increased a few hours after a meal. These results show the feasibility of the proposed system for monitoring the isoprene concentration in breath.

## 4. Conclusions

For the real-time monitoring of isoprene in breath, an analysis system based on ultraviolet-absorption spectroscopy (using a hollow optical fiber as a gas cell) was developed. The hollow optical fiber functioned as an extremely-small-volume gas cell with a long path that can detect minor components in the breath. Firstly, the measurement sensitivity of the system was evaluated by using nitric-oxide gas as a gas sample. The evaluation result showed that the system—using a laser-driven, high-intensity light source and a 3-m-long, aluminum-coated hollow optical fiber—could successfully measure nitric-oxide gas with a 50 ppb concentration. An absorption spectrum of a breath sample in the wavelength region of around 200–300 nm was measured with the proposed system, and the measured spectrum revealed the main absorbing components in breath as water vapor, isoprene, and ozone converted from oxygen by the radiation of ultraviolet light. The concentration of isoprene in breath was then estimated by multiple linear regression. It was shown that the proposed method enables real-time monitoring of isoprene during the exhalation of breath; in addition, it can measure isoprene variations in the daytime. Isoprene has been reported as a candidate for monitoring cholesterol metabolism and the proposed system, which is simple and low-cost, enables the real-time monitoring of isoprene in breath. Therefore, the proposed system can be a substitute for large-scale, sensitive equipment (such as a GC-MS system) and it is suitable for clinical applications. It is expected that the proposed system will contribute to further understandings of human metabolism in the near future.

## Figures and Tables

**Table 1:** Measured SNRs for three combinations of light source and fiber length.

Light Source, Fiber Length	SNR
LDLS, L = 3 m	42.1
LDLS, L = 1 m	19.4
D_2_ Lamp, L = 1 m	4.60
